# Maturity-associated variation in the body size, physical fitness, technical efficiency, and network-based centrality measures in young soccer players

**DOI:** 10.1038/s41598-023-34833-1

**Published:** 2023-05-11

**Authors:** Paulo Henrique Borges, Julio Cesar da Costa, Luiz Fernando Ramos-Silva, Vanessa Menezes Menegassi, Gibson Moreira Praça, Felipe Arruda Moura, Enio Ricardo Vaz Ronque

**Affiliations:** 1 Department of Physical Education, School of Sports, Federal University of Santa Catarina (UFSC), University Campus, Trindade, Florianópolis, Santa Catarina 88010-970 Brazil; 2 State University of Londrina (UEL), Londrina, Brazil; 3 State University of Maringá (UEM), Maringá, Brazil; 4 Universidade Federal de Minas Gerais (UFMG), Belo Horizonte, Brazil

**Keywords:** Psychology and behaviour, Bone development

## Abstract

This study aimed to observe the relationships between the maturity status on the network-based centrality measures of young athletes in small-sided soccer games (SSG). The study included 81 male players (14.4 ± 1.1 years). Measurements included height, sitting height, body mass, and bone age (TW3 method). The applied protocols were the following: Countermovement Jump (CMJ), Yo-Yo Intermittent Recovery Test Level 1 (YYIRT1), Repeated Sprints Ability (RSA), observational analysis of techniques, and interactions performed by players in SSG. The relationship between the set of evaluated variables within each maturity status was obtained from the correlational analysis of networks (P < 0.05). The maturity status explained a significant portion of the variance in body mass (η^2^ = 0.37), height (η^2^ = 0.30), sitting height (η^2^ = 0.30), and performance on the YYIRT1 (η^2^ = 0.08), CMJ (η^2^ = 0.14), and RSA (η^2^ = 0.13). No effect of maturity status on network-based centrality measures of young athletes was identified (P > 0.05). For the late maturity group, there was a correlation between the degree of centrality and physical growth indicators (r_mean_ = 0.88). For players with maturation “on time”, physical growth indicators relate to the degree of prestige (r_mean_ = 0.36). It is concluded that body size and bone age impact how late and on-time maturity groups interact within the match.

## Introduction

Soccer characterizes by the confrontation between two teams in an unpredictable, random, and complex context, where the individual interests of the players must be subordinated to the collective interests of the team for effective management of the game space^1^. Differences in players’ characteristics explain part of the large variability inherent to the game. Previous studies showed that playing position, age, and maturational status may impact players’ behaviors during game-related tasks in soccer^2–4^. Therefore, it is worth investigating whether individual characteristics impact performance indicators in young soccer to better select and develop talented players.

In line with this, there is an effort by the scientific community to develop studies that can help coaches and professionals involved with the modality to understand the idiosyncrasies related to players from the physical dimensions^5,6^, techniques^7,8^, tactics^9,10^, and psychological features^11,12^, facilitating eventual game strategies and methodological adjustments.

Adjusting training methods in soccer is complex due to the abovementioned multifactorial performance characteristic in the modality. Based on this, the literature suggests moving towards a multidimensional performance analysis^13^ to capture the inherent dynamics of the game better. This approach, followed in the current study, is expected to promote greater harmony between research, training, and competition^14^.

An alternative to help this intricate challenge is the social network analysis (SNA), which allows for identifying cooperative patterns among players at a “macro” structural level and also levels of tactical prominence of each player at a “micro” level^15,16^. Previous studies that used this method found that the quality of the team^17^, the playing position^18,19^, the partial result^20^, and chronological age^21^ influence to some extent the interaction of soccer players within their respective networks.

During this cooperative-interactive process, the tactical-technical actions performed by players within the game environment emerge from the unique combination of constraints imposed by the individual-environment-task triad^22,23^. At the individual constraint level, the subject’s characteristics that will condition their motor actions, such as muscle strength, flexibility, and body size, stand out. In turn, the restrictions of the environment concern the physical place where the soccer player is inserted in their practice, such as the context of small-sided games, which promotes an intense search for spaces and possession of the ball. Finally, the game’s objectives and rules can be considered task constraints. Thus, the mutuality between these aspects will provoke an adaptive motor response to respond to certain problem situations at the start^24^.

From this scenario, it appears that in the context of youth training, there is a need to investigate to what extent the status of maturity evidenced by the subject impacts the way players engage in tactical-technical actions within the match since the maturation process influences morphofunctional attributes of young soccer players^3,25,26^, and these characteristics can act as individual restrictors during decision-making in the game, with a consequent repercussion on motor responses and interactive choices within the match.

Additionally, most studies that analyzed the maturation process of young soccer players have adopted the assessment of individual and/or team performance from one or two dimensions, which may neglect the interaction between the different constraints that make up a tactical-technical scenario^13^. Collectively, this information suggests adopting multidimensional criteria to understand the impacts of different maturity statuses on the dimensions that make up the soccer game.

Thus, it is supposed that players who mature rapidly centralize the main actions throughout the match compared to their peers. This assumption is based on previous studies that indicated that early maturation favors better physical performance^27^, as well as triggers advances in the perceptual-cognitive level of young soccer players^28^. These advantages can contribute to accurate decision-making throughout the match, as they favor the execution of support movements for teammates, guarantee entry into free spaces, as well as help the perception of relevant signals in confrontation situations through capturing, recognizing, organizing, and understanding contextual information^29^.

Thus, the objectives of this study were: (I) to verify the relationships of the maturity status on the network-based centrality measures of young soccer players in small games; and (II) to identify the relative contributions of physical, anthropometric, and technical variables on the measures of centrality, from the different maturity status. Based on previous assumption, our initial hypothesis is that the influence of early maturation on the player’s physical performance can affect their centrality measures during the game, and that these measures are related to anaerobic performance, technical efficiency, and body size within each maturity status group.

## Methods

### Experimental design

In this cross-sectional study, data collection took place between September and October 2018 and occurred over four days^7^. On the first day, anthropometric measurements were conducted, followed by the performance tests; on the second day, specific skills tests, and then repeated sprint’s ability test were conducted; on day three, players had their wrists x-rayed; and on the last day, small-sided games were performed (GK + 3 vs. 3 + GK). Players were instructed to recover 48 h before the beginning of data collection, and the recovery time was 21 h between tests. Regarding the weekly training volume, the U-13 category participated on average in 2 weekly training lasting 120 min each. The U-15 category, in turn, trained on average 5.49 ± 0.49 weekly training units, lasting 120 min each.

### Participants

Eighty-one young male soccer players (14.37 ± 1.12 years; 58.03 ± 10.33 kg; 169.85 ± 10.04 cm) belonging to two soccer teams that compete at national and state levels^2^ participated in the study. Data collection occurred during the subjects’ competitive period, in which all athletes were in the middle of the competitive period. The following inclusion criteria were adopted: (I) train with one of the selected teams, (II) participate in official competitions for the club; and (III) present the free and informed consent form signed by parents or guardians, as well as the assent form. Subjects who presented musculoskeletal injuries during the evaluation period were excluded, and those who had not completed all project evaluations were excluded. The study was carried out in accordance with relevant guidelines and regulations, and it was approved by the Human Ethics Committee of the State University of Londrina (Proc. 2.650.232/2018).

### Anthropometry

Body mass, height, and sitting height were obtained using a digital scale Seca 813®, with a precision of 0.1 kg, and a portable stadiometer, Harpenden®, with a precision of 0.1 cm, according to procedures described in the literature^30^.

### Chronological age and bone age

The chronological age of the players was obtained in a centesimal way from the difference between the date of birth and the date of the anteroposterior radiograph of the hand and wrist. Players were required to take an anteroposterior radiograph of the hand and wrist in a specialized laboratory to obtain an x-ray of the left hand and wrist. Subsequently, the Tanner-Whitehouse 3 method was adopted to classify 13 bones of the left hand and wrist according to their stage of development. Based on these scores, it was used a sex-specific table to convert scores into bone age^31^. A radiologist performed the radiography, and bone age assessments was conducted by a trained researcher.

To test the reliability of the bone age assessment, the same rater randomly reassessed 20 hand and wrist radiographs two weeks after the first assessment. The intraclass correlation coefficient found was 0.97, and the intra-observer error was 0.26 years. The classification of the maturity status of young athletes took place in two stages. Initially, the difference between the bone age and the chronological age of the soccer players was obtained. Based on this difference, the sample was divided into early (difference > 1 year), “on time” (difference ± 1 year), and “late” maturity players (difference < − 1 year), according to the criteria suggested by the literature^4^.

### Physical fitness

The Yo-Yo Intermittent Recovery Test level 1 was used to estimate the individual’s ability to perform intense exercise repeatedly. The objective of the test was to run 20 m from a cadence pre-established by audio, with 10 s of rest every 40 m walked^32^. The final score was expressed by the maximum distance in meters covered by the athlete. Lower limb muscle power was estimated based on a vertical jump proposed by Bosco, Luhtanen, and Komi^33^ called Counter Movement Jump (CMJ), performed on a contact platform (Hidrofit®) connected to the computer. The subjects performed 3 jumps, with a one-minute interval between attempts, with only the jump with the best performance being computed. The anaerobic performance of young soccer players was evaluated using the Repeated Sprints Ability (RSA) test proposed by Rampinini et al.^34^. This protocol consists of performing six runs of 40 m each, separated by 20 s of recovery. The running time was recorded using a set of photocells (Multisprint Full®) connected to the computer. The time spent during the six runs was computed, in seconds, for the athlete’s score.

### Small-sided games protocol

To evaluate the tactical-technical actions, each player was filmed in the game GK + 3 vs. 3 + GK (goalkeeper + 3 players vs. 3 players + goalkeeper), being this a small-sided game in a field of 36 m by 27 m during 2 periods of 4 min each, with a 1-min break. The camcorder (Cassio® model EX-10) was located 6 m above and to one side of the pitch long axis at a distance of 15 m from the pitch. The official rules of the modality were adopted, including the offside rule, as described in Borges et al.^2^.

### Social network analysis and technical efficiency

From footage of players in the game GK + 3 vs. 3 + GK, an observational protocol^35^ was used to analyze the interactions carried out in the small-sided games and to observe the technical actions performed by the young soccer players. Regarding social network analysis (SNA), the execution of a pass between two players was adopted as a criterion for interaction between them^36^. The Social Network Visualizer® software (SocNetV 1.9 © 2005–2015 by Dimitris V., Kalamaras) was used to graphically visualize the interactions between the players and obtain the following information: degree centrality, indicating the number of passes made by the player within the network; closeness centrality, which in sports context may be understood as a measure of the ability of a node (player) to access or send information to other nodes on the network; degree of prestige, concerning the number of passes that the player receives within the network; and proximity prestige, expressing the geodesic distance of other teammates from a specific player, suggesting that a player with high proximity prestige values may receive more passes from teammates in the case of a pass^37^.

The individual technical actions of each subject were obtained from the protocol proposed by Gréhaigne, Mahut, and Fernandes^38^: conquered ball (CB), which refers to the action of re-conquering the ball through interception, direct recovery over the opponent, or after an unsuccessful shot on goal; offensive balls (OB), considered a pass to a teammate that puts pressure on the other team and, most of the time, leads to a shot on goal; successful kick (successful shot—SS), when the shooting action ends in a goal or ball possession returns to the attacking team; and lost ball (LB), which occurs when the player loses the ball to an opponent without having finished on goal. A specific equation was used from these indicators to obtain the technical efficiency index: (CB + OB + SS)/(10 + LB). The analysis was performed using the Lince® software (version 1.4)^39^.

To assess the quality of the observed data, intra- and inter-rater reliability analyses were used. In this sense, intraclass correlation coefficients above 0.82 were obtained, and both assessments revealed good/excellent reliability.

### Statistical analysis

The relationships of maturity status on indicators of body growth, physical performance, and measures of the centrality of young soccer players were tested using multivariate analysis of variance (MANOVA) after validating the assumptions of data normality and homogeneity of data variances-covariances, applied to the Kolmogorov–Smirnov tests (p > 0.05 for all groups) and M of Box for the purposes mentioned above. Additionally, the multivariate analysis of covariance (MANCOVA) was applied for variables that differed significantly and adjusted for chronological age. The above analyses were processed using the SPSS software (v. 23, IBM SPSS, Chicago, IL), considering the significance set at 5%.

Pearson’s correlation coefficient and the correlational analysis of networks were applied using the “qgraph” package to verify the degree of relationship between the set of study variables based on the maturity status. Correlational analysis of networks constitutes an essential methodological tool to investigate complex patterns of interaction between variables^40^, which allows the visualization of how the different dimensions that comprise sports performance interact between the different maturity statuses. In this analysis, a vertex represents each variable, and links form the relationship between them (correlation). In presented graphs, green lines indicate positive correlation, and red lines are negative correlations. The representation of connections with weights was adopted; that is, the thickness of the connection is associated with the strength of the correlation.

The importance of each variable in determining the structure of the network was observed from the following centrality indicators: strength, which is obtained from the sum of the strength of the connections that the vertex receives; betweenness, providing information about the importance of a vertex among other vertices, that is, a vertex with high connectivity plays a fundamental role in the network, as it represents the shortest path between two other vertices; and closeness, a metric used to quantify the relationship of a vertex to all other vertices, where high degrees of proximity indicate a short distance between the other vertices and symbolizes that any change in this variable quickly affects other parts of the network^40^. This analysis was processed in R (version 4.0.0) and RStudio (version 1.2.5042) software.

## Results

Table 1 shows the characteristics of the players by maturity status. Early players had higher bone age, body mass, height, and sitting height compared to the on-time and late groups (p < 0.05), even controlling for chronological age (Pillai’s Trace = 0.77; F = 6.47; p = 0.01; η^2^ = 0.38). Regarding the network-based centrality measures of young soccer players, there was no difference between the maturity status (p > 0.05).

**Table 1:** Anthropometric, physical, technical, and tactical prominence characteristics of young soccer players grouped by maturity status: mean, standard deviation (SD), and MANOVA results to compare maturational groups; and adjusted mean for chronological age, standard error (SE), and MANCOVA results with chronological age as a covariate (N = 81).

	MANOVA						MANCOVA (age as covariate)						
	Late (L) (n = 7)	On time (OT) (n = 57)	Early (E) (n = 17)	F	P	η^2^	Late (L) (n = 7)	On time (OT) (n = 57)	Early (E) (n = 17)	F	P	η^2^	Post Hoc
	Mean (SD)	Mean (SD)	Mean (SD)				Mean (SD)	Mean (SD)	Mean (SD)				
CA (years)	13.46 (1.19)	14.48 (1.14)	14.39 (0.86)	2.70	0.07	0.06							
BA (years)	11.80 (0.78)	14.71 (1.26)	15.72 (0.77)	28.92	0.01	0.42	12.51 (0.19)	14.62 (0.63)	15.70 (0.11)	236.88	0.01	0.90	E > OT&L; OT > L
BM (kg)	39.40 (5.02)	58.36 (8.76)	64.60 (7.59)	22.99	0.01	0.37	43.73 (2.18)	57.76 (0.70)	64.45 (1.28)	75.84	0.01	0.74	E > OT&L; OT > L
Height (cm)	153.08 (8.71)	170.36 (8.76)	175.05 (7.40)	16.91	0.01	0.30	157.40 (2.51)	169.77 (0.81)	174.93 (1.47)	46.58	0.01	0.64	E > OT&L; OT > L
SH (cm)	78.21 (4.53)	88.18 (5.15)	90.64 (3.74)	16.75	0.01	0.30	80.53 (1.44)	87.86 (0.46)	90.55 (0.84)	45.62	0.01	0.64	E > OT&L; OT > L
YYIRT (m)	811.29 (186.52)	1061.05 (290.08)	1158.00 (272.73)	3.80	0.02	0.08	924.87 (98.78)	1045.85 (31.76)	1158.90 (57.97)	13.28	0.01	0.34	E > L
CMJ (cm)	27.04 (1.76)	31.28 (4.57)	33.60 (2.87)	6.39	0.01	0.14	28.27 (1.53)	31.11 (0.49)	33.60 (0.90)	11.22	0.01	0.30	E > L
RSA (sec.)	50.24 (2.44)	47.51 (2.40)	46.61 (1.97)	6.04	0.01	0.13	48.79 (0.60)	47.69 (0.19)	46.62 (0.35)	48.98	0.01	0.65	E&OT > L
TE (score)	0.45 (0.27)	0.48 (0.28)	0.46 (0.22)	0.05	0.94	0.01							
DC (%)	0.37 (0.08)	0.32 (0.10)	0.33 (0.09)	1.05	0.35	0.02							
CC (d)	1.79 (0.90)	1.89 (0.99)	1.59 (0.83)	0.66	0.52	0.01							
DP (%)	0.36 (0.09)	0.31 (0.09)	0.33 (0.06)	1.04	0.35	0.02							
PP (d)	0.28 (0.08)	0.26 (0.12)	0.29 (0.14)	0.25	0.77	0.01							

*CA* chronological age, *BA* bone age, *BM* body mass, *SH* sitting height, *YYIRT* Yo Yo intermittent recovery test, *CMJ* counter movement jump, *RSA* repeated sprints ability; *TE* technical efficiency, *DC* degree centrality, *CC* closeness centrality, *DP* degree prestige, *PP* proximity prestige.

**Figure 1:**
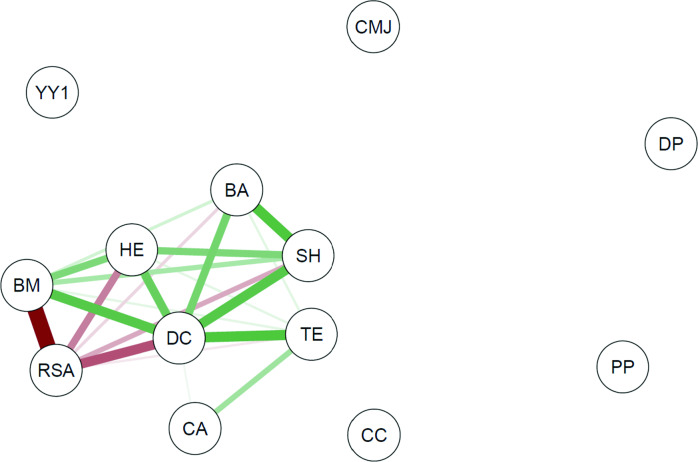
Network of correlations between centrality in offensive actions, technical efficiency, growth indicators, bone age, and physical performance of young soccer players with “late” maturation. *BA* bone age, *BM* body mass, *HE* height, *SH* sitting height, *YY1* Yo Yo intermittent recovery test, *CMJ* counter movement jump, *RSA* repeated sprints ability, *TE* technical efficiency, *CA* chronological age, *DC* degree centrality, *CC* closeness centrality, *DP* degree prestige, *PP* proximity prestige.

Figure 1 presents the correlation network of several variables among late-maturity players. Making connections within the game, represented by the degree of centrality, was related to the athletes’ bone age (BA; r = 0.86), as well as body mass (BM; r = 0.89), height (HE; r = 0.87), sitting height (SH; r = 0.89), repeated sprints ability (RSA; r = − 0.44), and technical efficiency (TE; r = 0.90). No significant relationships were observed for the other network-based centrality measures in offensive actions with the set of variables of the statistical model.

**Figure 2:**
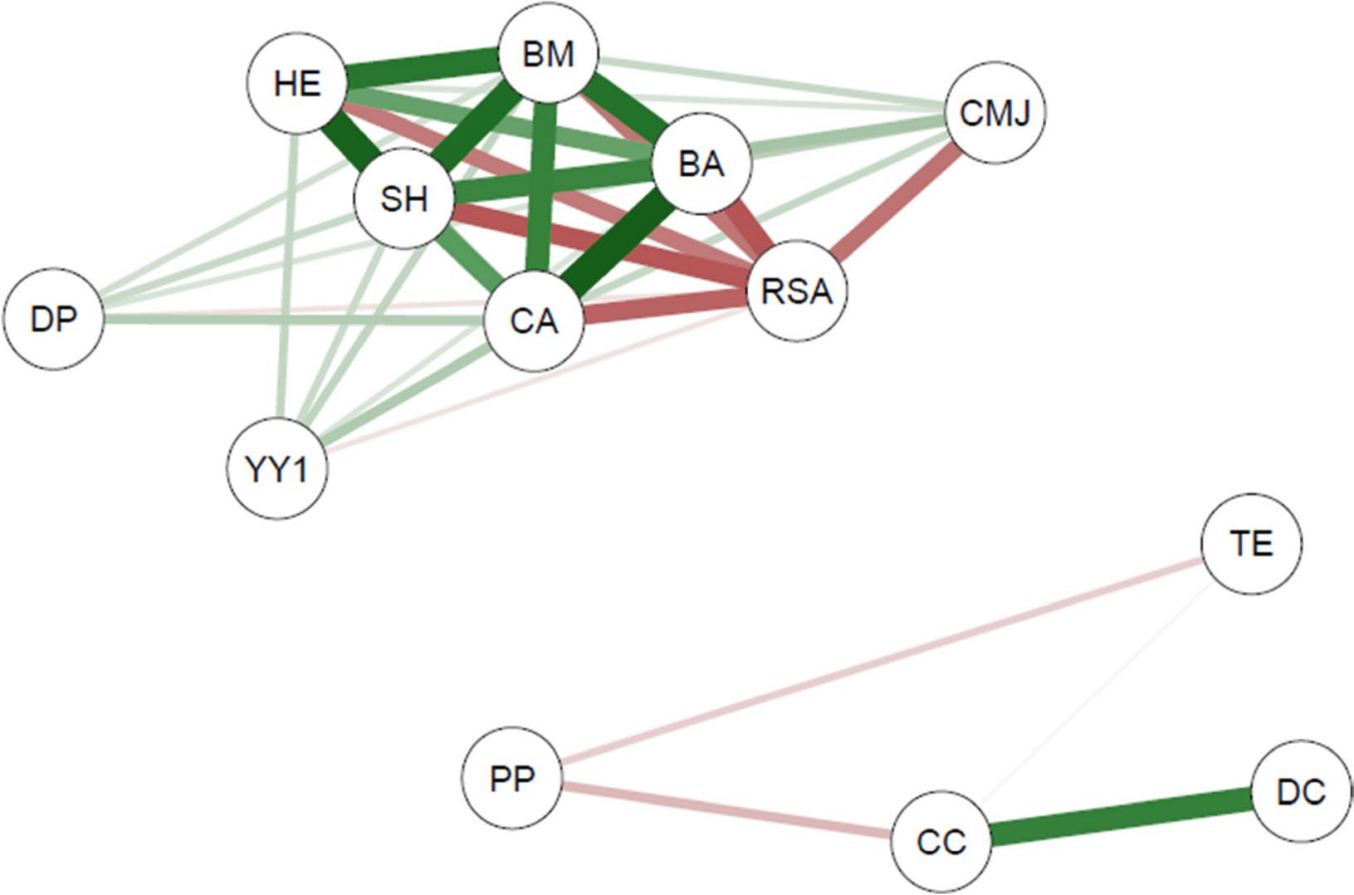
Network of correlations between centrality in offensive actions, technical efficiency, growth indicators, bone age, and physical performance of young soccer players with maturation "on time". *BA* bone age, *BM* body mass, *HE* height, *SH* sitting height, *YY1* Yo Yo intermittent recovery test, *CMJ* counter movement jump, *RSA* repeated sprints ability, *TE* technical efficiency, *CA* chronological age, *DC* degree centrality, *CC* closeness centrality, *DP* degree prestige, *PP* proximity prestige.

The variables analyzed from the players with maturity status “on-time” (Fig. 2) showed correlations between proximity prestige and technical efficiency (r = − 0.37). The degree of prestige correlated with bone age (r = 0.34), body mass (r = 0.35), sitting height (r = 0.37), and repeated sprints (r = − 0.32).

**Figure 3:**
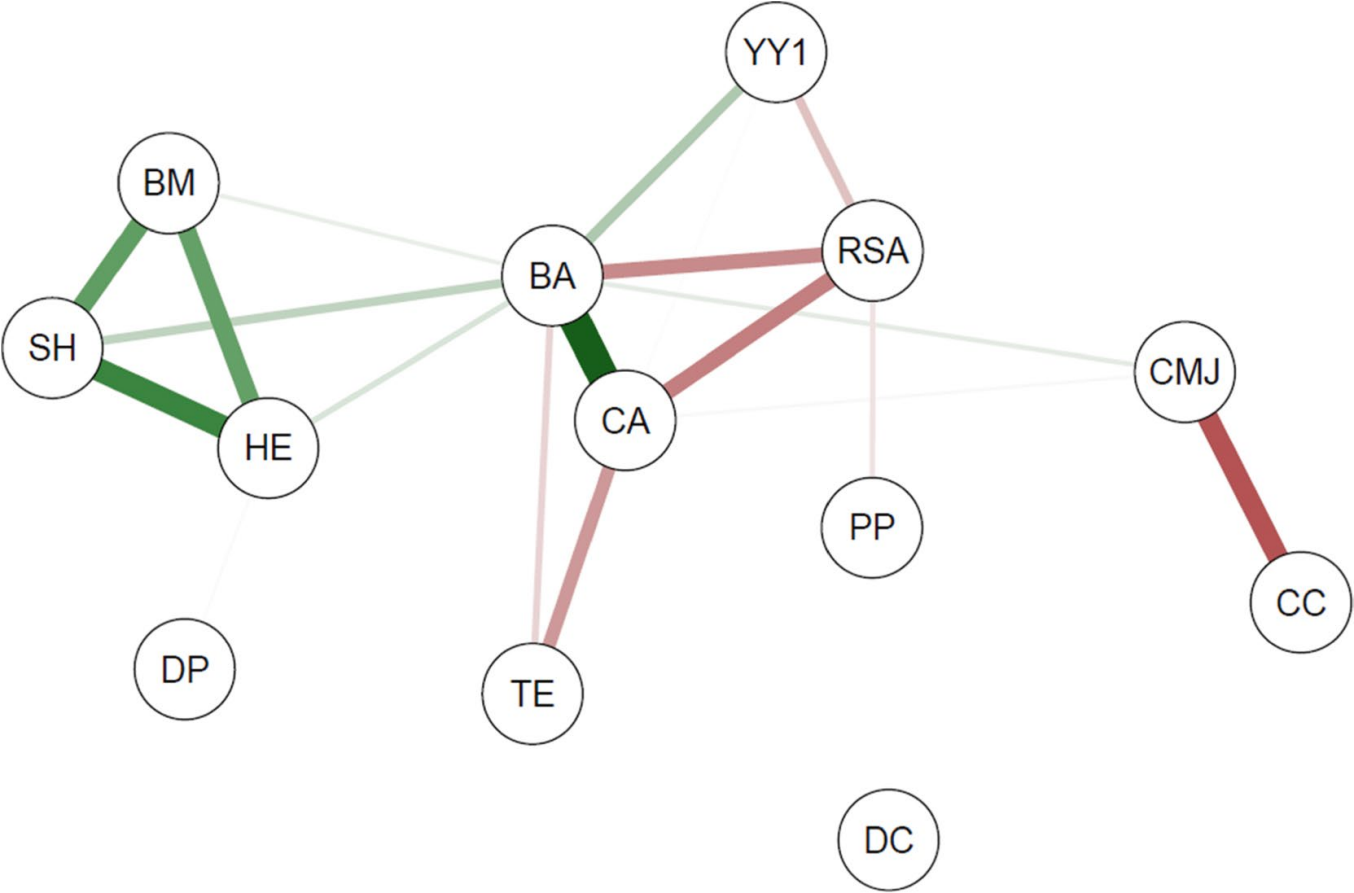
Network of correlations between centrality in offensive actions, technical efficiency, growth indicators, bone age, and physical performance of young soccer players with “early” maturation. *BA* bone age, *BM* body mass, *HE* height, *SH* sitting height, *YY1* Yo Yo intermittent recovery test, *CMJ* counter movement jump, *RSA* repeated sprints ability, *TE* technical efficiency, *CA* chronological age, *DC* degree centrality, *CC* closeness centrality, *DP* degree prestige, *PP* proximity prestige.

Figure 3 symbolizes the relationships observed between the different variables used in this study for the group with early maturation. For the referred group, the physical growth indicators are only correlated with each other, and these variables did not present correlations with the centrality measures in offensive actions (p > 0.05). Bone age relates to technical efficiency (r = − 0.55), proximity prestige relates to repeated sprints (r = − 0.52), and degree of prestige relates to Counter-movement Jump performance (r = 0.77).

**Figure 4:**
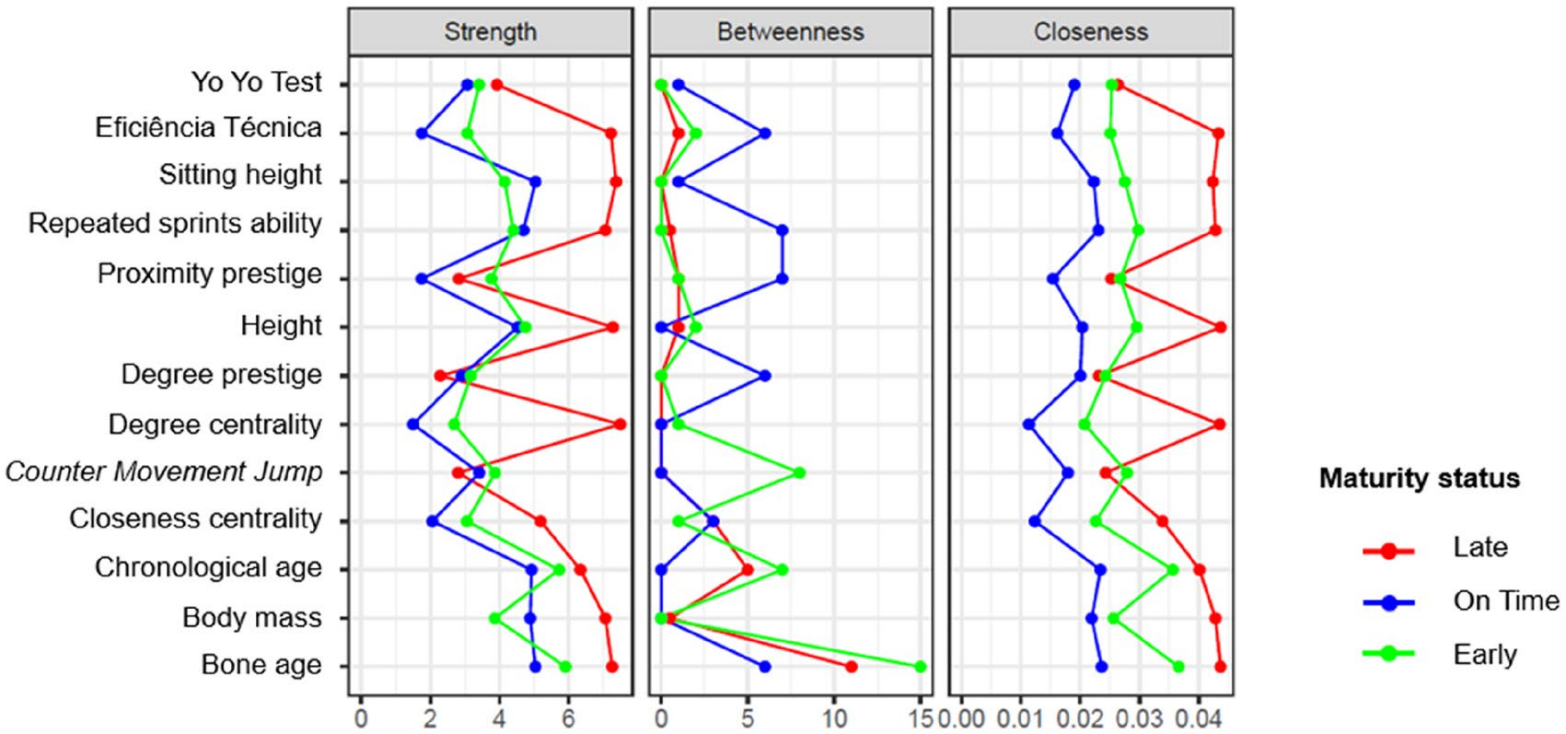
Strength, betweenness and closeness of the set of variables analyzed in the network of relationships. Values were expressed in the z score.

The centrality indices of each variable in the correlation network are shown in Fig. 4. For the late group, the degree of centrality, sitting height, height, technical efficiency, and bone age showed the highest connection forces. In contrast, bone age exhibited the highest mediation value in the network of variables (betweenness). Technical efficiency, height, degree of centrality, and bone age were the main variables that, if modified, can modify the others in the correlation network.

Concerning the “on-time” group, sitting height, chronological age, body mass, and bone age were the attributes with the highest strength values. Additionally, the Repeated Sprints Ability and proximity prestige variables showed higher values to mediate the relationships between the domains evaluated (betweenness). The Repeated Sprints Ability test, chronological age, and bone age showed the smallest distances between the variables analyzed (closeness).

Regarding the centrality values of the “early” group, it was observed that bone age and chronological age showed the highest strength values; bone age also showed the greatest ability to mediate relationships between two or more variables. Finally, bone age and chronological age showed centrality in the network in question, being variables with smaller distances between the other attributes of the model.

## Discussion

This study verified the relationships of the maturity status on network-based centrality measures of young soccer players in small-sided games and identified the relative contributions of physical, anthropometric, and technical variables on centrality measures from the different maturity statuses. Contrary to the initial hypotheses of the study, the main results showed that the status of maturity did not influence the centrality measures based on the interactions performed by the players in a reduced game. However, when analyzed from a multidimensional scenario, measures of centrality in offensive actions relate differently to anaerobic performance, technical efficiency, and body size within each maturity status.

The subjects who presented early maturation showed greater anthropometric measurements compared to their peers, corroborating previous studies in soccer^41,42^, basketball^43^, and handball^44^. Such an explanation lies in the fact that, although growth is a universal and intense biological mechanism during adolescence, the combination of genetic, hormonal, nutritional, and social factors cause individuals to vary among themselves in the rate of regulation of progress toward growth mature state.

Moreover, differences in body mass and height among maturational groups could be related specifically to the increase in hormone concentration around puberty^45^. For example, the growth hormone (GH) concentration acts directly upon height, and it has been observed to double its levels during APHV^46^. Besides that, it is also expected that early-maturing athletes present higher sitting height measures compared to late-maturing athletes since body growth is processed earlier in the cranial, proximal, and then general structures^47^.

Additionally, it was observed that early-maturity players presented better aerobic, anaerobic, and jump performance. The advance in the maturity status associates with an increase in the number of cells in the body and a progressive improvement in energy production, resulting in better enzymatic efficiency necessary for the demands related to sport^48,49^. This information corroborates with Falgairette et al.^50^ and Arruda et al.^51^, who found significant correlations between anaerobic performance and salivary testosterone levels.

In line with this, a systematic review with meta-analysis produced by Albaladejo-Saura et al.^52^ identified that early athletes presented better results in tests in which there were related to the ability to produce power and strength, which may be associated with the increase of testosterone hormone and its effects on muscle mass gain. However, in the referred meta-analysis, the authors did not identify differences among different maturational groups in the Yo-Yo Test, concluding that the aerobic performance seems to be influenced by training variables^52^. Considering the controversial results of the current study with the meta-analysis in terms of aerobic performance, further research to address this issue is recommended.

Thus, it is observed that physical performance plays a vital role in youth training, as it allows the involvement and maintenance of players in tactical-technical tasks during different game situations. However, the influence of morphological and functional variables on the involvement in tactical-technical actions assumed different features among different maturational statuses. In the present study, for the late-maturation group to interact more in the small-sided games, the subjects needed to demonstrate greater technical efficiency, combined with greater body size and better anaerobic performance, suggesting that players with late maturation can compensate for the low performance in physical tests with high technical efficiency, being considered references for cooperative actions among teammates, transmitting security for the maintenance of ball possession^53^.

These results also reinforce the role of anaerobic metabolism during the execution of tactical and technical actions by young soccer players in small-sided games, as the Counter-movement Jump test evaluates components that require the referred energetic pathway for its manifestation. Likewise, the restrictions imposed by the small format of the game environment require permanent changes of direction, starts, and jumps from players, which are movements that also recruit anaerobic metabolism^54^. Therefore, it appears that the late subjects in the maturation process, who performed better in these tasks, could perform more passes and interact more with their peers.

On the other hand, concerning players with a status of maturity on time, the indicators of physical growth only contributed to the receiving of passes within the small-sided games. This can be explained by the fact that the increase of one unit of lean mass (in kilograms) corresponds to an improvement of 0.25 s in the running time^55^, which potentially favors the performance of quick movements that guarantee the entry into spaces conducive to offering support to the ball carrier, with subsequent increase in the degree of prestige observed.

Regarding the results found for the group with early maturation, body size was not associated with centrality measures in the network, revealing that a better anaerobic performance corroborates a greater distance from teammates. This result aligns with Clemente et al.^53^, who identified an association between low fatigue levels and movement toward teammates. One factor that may help explain the discrepancy in these findings is the lack of control over the playing position in the statistical analyses. According to reports by Boone et al.^56^, forwards are faster in short runs than goalkeepers, defenders, and midfielders. Furthermore, goalkeepers and defenders performed better in the vertical jump than midfielders.

Thus, it is believed that early maturity players have advantages related to determinants of anaerobic performance, such as greater amounts of lean mass, availability of substrate in muscle cells, and greater neuromotor control^57,58^, and these characteristics can favor the search for free spaces in the field of play, as well as rapid mobility movements towards the opponent’s field, contributing to a distance of the player about the teammate in possession of the ball.

These relationships are expected in the soccer game, as mentioned by Guilherme, Garganta, and Graça^59^ and Praça et al.^60^. During the situations arising from the match, players are tactically required to decide “what to do” to subsequently choose an intelligent and creative motor response that satisfies the demands of the referred problem. However, most studies investigating the impact of maturation on the technical actions of young soccer players used assessment instruments in controlled situations, with the absence of complex decision-making processes^3,61,62^, making it difficult to compare the results found with other previous investigations published in the literature.

Considering also the information that early maturity players are predominantly selected in youth soccer teams due to their physical and anthropometric qualities^25,27^, it is reasonable to infer that late athletes selected and who remain in the sports environment compensate for their morphofunctional weaknesses by technically and tactically equaling the other players, performing a valuable and efficient management of the game space for the team.

The results found in the present study showed the complexity inherent to understanding the role of physical growth and biological maturation in the variation of physical, technical, and tactical indicators. Physical growth indicators and bone age directly affect interactions in reduced games, especially for late and on-time groups. As young soccer players mature, body size plays a limited role in the interactions established in the game, with other performance variables beginning to occupy a prominent role, such as functional capabilities and technical efficiency.

It should be noted that soccer is a multifactorial sport. So, the variation portion not explained by the anthropometric and maturational indicators inserted in the statistical models presented in this work may come from factors in the social environment, such as the teaching-training method adopted^9^, the momentary result of the match^63,64^, and the quality of the opposing team^65^.

The small number of categories investigated can be understood as a limitation of the study, as it makes it impossible to understand the impact of the maturity status on tactical-technical actions in young people from other categories. Moreover, the small sample size requires caution, especially when using correlation and multivariate models in statistics. Another study limitation may be the use of a small-sided game format (GK + 3 vs. 3 + GK), which made it difficult to expand these results to official matches. However, the use of an x-ray of the hand and wrist to obtain bone age, and also the application of real game situations to assess tactical and technical metrics (instead of isolated measures of skills) may be considered strengths of this paper with young athletes. The results allow coaches and professionals involved with soccer players aged between 12 and 15 to understand how the different variables relate to individuals with different maturity statuses. Future research may wish to comprehend how maturational statuses impact performance variables inside other game formats and official matches.

## Conclusion

It is concluded that the status of maturity has a low influence on measures of centrality based on the interactions of young soccer players in offensive actions. Late-maturing athletes who interacted more in the game presented better technical efficiency, body size, and also anaerobic performance. For the on-time maturing athletes, higher measures in body size were associated with receiving the ball from teammates. For the early maturity athletes, body size and maturation didn’t relate to the centrality measures in the game.

For future studies, a longitudinal research design involving a multidimensional scenario is suggested to identify the consistency of the results found in the present study with other populations and different competitive levels.

## Data Availability

The study results are presented clearly, honestly, and without fabrication, falsification, or inappropriate data manipulation. All data are fully available uppon email request to the corresponding author.
